# Identification of DNA methylation biomarkers with potential to predict response to neoadjuvant chemotherapy in triple-negative breast cancer

**DOI:** 10.1186/s13148-021-01210-6

**Published:** 2021-12-18

**Authors:** Braydon Meyer, Samuel Clifton, Warwick Locke, Phuc-Loi Luu, Qian Du, Dilys Lam, Nicola J. Armstrong, Beena Kumar, Niantao Deng, Kate Harvey, Alex Swarbrick, Vinod Ganju, Susan J. Clark, Ruth Pidsley, Clare Stirzaker

**Affiliations:** 1 Epigenetics Research Laboratory, Genomics and Epigenetics Theme, Garvan Institute of Medical Research, 384 Victoria Street, Darlinghurst, Sydney, NSW 2010 Australia; 2 Molecular Diagnostics Solutions, CSIRO Health and Biosecurity, New South Wales, North Ryde, 2113 Australia; 3 St. Vincent’s Clinical School, UNSW Australia, Sydney, NSW 2010 Australia; 4 Department of Mathematics and Statistics, Curtin University, Perth, WA 6103 Australia; 5 Hudson Institute of Medical Research, Clayton, VIC 3168 Australia; 6 Monash Health Pathology, Monash Health, Victoria, 3168 Australia; 7 Monash University, Melbourne, VIC 3168 Australia; 8 Cancer Research Theme, Garvan Institute of Medical Research, Sydney, NSW 2010 Australia

**Keywords:** DNA methylation, Methylome, Epigenetics, Triple-negative breast cancer, Neoadjuvant chemotherapy

## Abstract

Neoadjuvant chemotherapy (NAC) is used to treat triple-negative breast cancer (TNBC) prior to resection. Biomarkers that accurately predict a patient’s response to NAC are needed to individualise therapy and avoid chemotoxicity from unnecessary chemotherapy. We performed whole-genome DNA methylation profiling on diagnostic TNBC biopsy samples from the Sequential Evaluation of Tumours Undergoing Preoperative (SETUP) NAC study. We found 9 significantly differentially methylated regions (DMRs) at diagnosis which were associated with response to NAC. We show that 4 of these DMRs are associated with TNBC overall survival (*P* < 0.05). Our results highlight the potential of DNA methylation biomarkers for predicting NAC response in TNBC.

**Supplementary Information:**

The online version contains supplementary material available at 10.1186/s13148-021-01210-6.

## Introduction

Triple-negative breast cancer (TNBC) represents ~ 15–20% of all breast cancers and compared with non-TNBC is associated with a higher risk of disease recurrence after treatment and shorter overall survival [1]. Neoadjuvant chemotherapy (NAC) is typically applied in the TNBC setting, and the degree of pathological response to NAC correlates with long-term prognosis [2]; 30–40% of TNBC patients achieve a pathological complete response (pCR), associated with a favourable outcome, while patients with a partial or lack of response (non-responder, nR) have a higher risk of relapse and poor prognosis [3]. Biomarkers of NAC response hold the potential to determine which patients will best respond to treatment and identify those patients who will fail to achieve a pCR, allowing personalised chemotherapeutic decision-making approaches within the clinic and avoiding unnecessary toxicity by ineffective treatment. However, there are no reliable methods to date that accurately predict response to NAC.

Epigenetic modifications of tumour DNA, including DNA methylation, are showing widespread promise as molecular biomarkers of disease and treatment response. In this study, we aimed to identify a DNA methylation signature in tumours predictive of response to NAC in TNBC patients.

## Material and methods

### Clinical samples

**Fig. 1:**
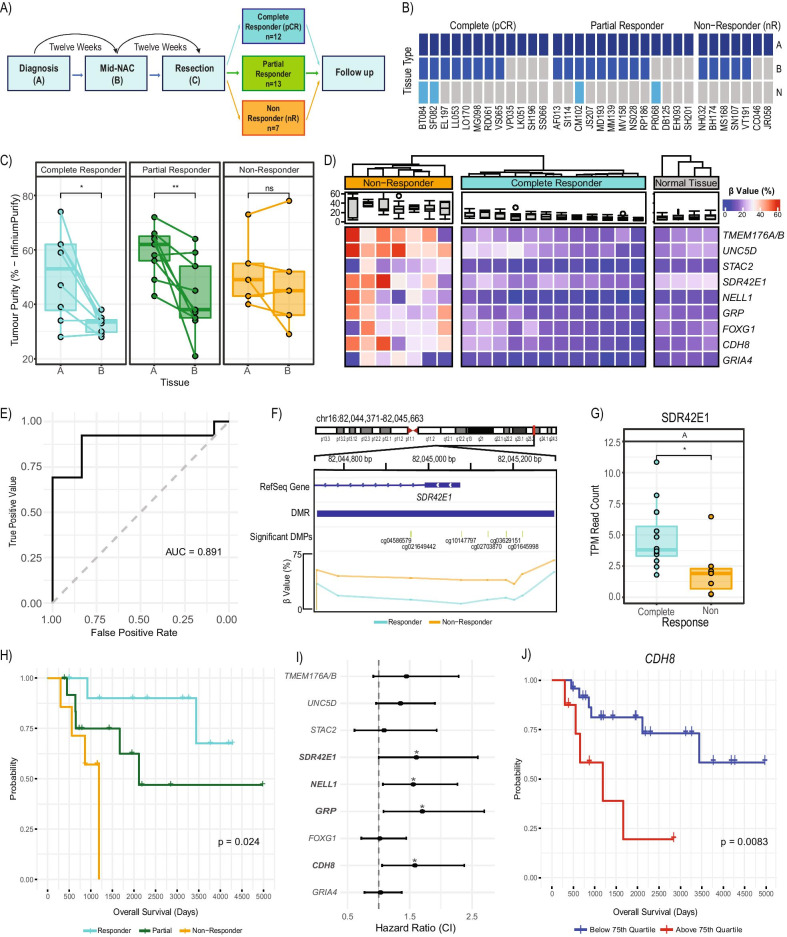
DNA methylation associated with response to NAC and patient survival in the SETUP study. **A** Overview of biopsy sample collection in the SETUP study. **B** Sample availability per patient at diagnosis and mid-NAC for DNA methylation profiling. **C** Boxplot of tumour purity estimated from DNA methylation in paired samples between biopsies A and B, shows that after 12 weeks of treatment complete and partial responders show an average 18% (*P* = 0.012) and 19.4% (*P* = 0.0084) reduction in tumour purity respectively. **D** Dendrogram and heatmap of the 9 significant response-DMRs (∆β > 10%, *FDR* < 0.1) found when comparing complete responders (*n* = 12) against non-responders (*n* = 7), with normal breast methylation data shown for reference (*n* = 4). **E** Receiver operating characteristic (ROC) curve showing the ability of the 9 response-DMRs to distinguish complete (*n* = 12) from partial responders (*n* = 13) on all diagnostic samples (biopsy A, AUC = 0.891). **F** Schematic of the *SDR42E1* gene promoter showing location of the response-DMR, individually significant probes and β values averaged within response group (pCR *n* = 12 and nR *n* = 7). **G** Boxplot showing significant differential expression of response-DMR *SDR42E1* (biopsy A, Welch t-test, *P* < 0.05). Survival analysis on all 9 response-DMRs was undertaken on the entire SETUP cohort at diagnosis (*n* = 32). **H** Kaplan Meier plot of overall survival stratified by patient response (Log-rank test, *P* = 0.024). **I** Forest plot showing the Cox hazard ratios (± 95% CI) for overall survival for each response-DMRs. **J** Kaplan Meier plot of overall survival for patients in the highest quartile of methylation for response-DMR *CDH8* (top 25%, red) versus the rest of the cohort (bottom 75%, blue), (HR = 1.58 (CI: 1.06, 2.38), Log-rank test, *P* = 0.0083)

Sequential Evaluation of Tumours Undergoing Preoperative chemotherapy (SETUP) study cancer samples were obtained from Monash and Peninsula Health (Melbourne, Australia). Women with locally advanced TNBC (T1-T3, N0-N3, MO), age > 18, were invited to participate in the SETUP study and were consented for the collection of imaging data and biological specimens for biomarker analysis. Women enrolled in the observational study received NAC with 12 weeks of FEC100 (fluorouracil 500 mg/m^2^, epirubicin 100 mg/m^2^, and cyclophosphamide 500 mg/m^2^), and 12 weeks of docetaxel (100 mg/m^2^; Fig. 1A). All patients underwent a clinical examination, mammography, ultrasound, breast MRI, and tumour core biopsy at diagnosis (prior to NAC, biopsy A) and all assessments and additional core biopsies were repeated after four cycles of chemotherapy (mid-NAC, biopsy B) and again at completion of the treatment regime (resection, biopsy C) (see Additional file 1 for further study details). Response to NAC was confirmed by PET, CT, and histology, with patients achieving a pathological complete response (pCR) having no evidence of tumour. Patients were considered non-responders (nR) if they showed no evidence of tumour reduction, or a partial responder if their tumour reduced in size but was not entirely eradicated. A total of *n* = 63 samples from 32 TNBC patients were acquired for analysis. The SETUP study was approved by the Human Ethics Research Committee and the Monash Medical Centre (ANZCTR.org.au clinical trials identifier: ACTRN12605000588695; HREC/SETUP/03169A0) as described in Alamgeer et al. [4].

### DNA methylation profiling

DNA was extracted from biopsy samples with the DNeasy kit (Qiagen) as previously described [5]. DNA methylation was quantified using the Illumina Infinium HumanMethylationEPIC BeadChip (Illumina, CA, USA) (EPIC arrays) using the manufacturer’s standard protocol. The EPIC array data was processed as previously described [6] (Additional file 1). The resulting dataset comprised 804,805 CpG sites.

Global methylation levels were calculated from repetitive elements targeted by the array using the *REMP* package (version 1.10.1). Genome-wide differential methylation analysis was performed on biopsies prior to NAC (biopsy A) between patient samples from the extreme ends of response, i.e., those that achieved a pathological complete response (pCR, *n* = 12) against those that did not respond to treatment (nR, *n* = 7). Partial responders (*n* = 13) were excluded from the initial discovery analysis to focus on the extremes of response (pCR and nR), as analysis of phenotypic extremes is a common approach in genomic studies to achieve greater statistical power. We included partial responders in secondary analyses to ensure that all treatment responses were considered. We used the *limma* package to identify differentially methylated probes (DMPs) between these response groups with adjusted p-value cut-off of FDR < 0.1. The R package *DMRcate* was used to identify differentially methylated regions (DMRs) i.e., response-DMRs, with a DMR p-value cut-off of FDR < 0.1 and a ∆β of >  = 10% (Additional file 1). *pROC* was used for the prediction of response in complete and partial responder samples utilising all significant DMRs.

### Survival analysis

Survival analysis was carried out using Log-rank tests and Cox proportional hazards model as implemented in the R *survival* package using overall survival data in the SETUP cohort (*n* = 32 diagnostic biopsies). There were 11 events within this dataset and significance was defined at *P* < 0.05. Package details and additional methods for all bioinformatic analysis can be found in Additional file 1.

## Results

To identify if DNA methylation alterations are associated with NAC response, we performed a genome-wide methylation analysis of TNBC samples from the SETUP neoadjuvant clinical study [4]. Biopsies (total *n* = 63) were taken at three timepoints: diagnosis (A, *n* = 32 tumour, *n* = 4 normal adjacent) and mid-chemotherapy (B, *n* = 22) followed by total excision post-chemotherapy (C, *n* = 5), (Fig. 1A and B). Patients were classified as: pathological complete responders (pCR, *n* = 12) where there was no evidence of tumour remaining post-chemotherapy (confirmed by PET, CT, and histology); partial responders (*n* = 13) where there was a reduction in tumour size from baseline; and non-responders (nR, *n* = 7) where tumour size was unchanged or progressed through chemotherapy [4] (Additional file 3: Table S1 & S2). We performed DNA methylation profiling of the SETUP biopsy samples using the EPIC arrays, resulting data underwent processing and quality control as detailed in Additional file 1. SNP probes on the array showed that all the samples clustered by patient as expected (Additional file 2: Fig S1).

First, we used cellular deconvolution analyses of the methylation data to quantify tumour purity and cell-type composition of each biopsy to inform further analyses. We estimated the epithelial content (representing tumour purity) using two approaches (*InfiniumPurify* & *EpiDISH*, Additional file 1) and found good agreement between the two (Additional file 2: Fig S2a). Whilst there were no significant differences in tumour purity between the responder groups at diagnosis (biopsy A, Additional file 2: Fig S2b), we observed a decrease in tumour purity in response to chemotherapy (biopsy A vs B) in complete (*n* = 8 pairs, paired Welch t-test, *P* = 0.012) and partial responders (*n* = 9 pairs, paired Welch t-test, *P* = 0.0084), which was absent in non-responder samples (Fig. 1C). *EpiDISH* also estimated other cell types including immune and fibroblast contents (Additional file 2: Fig S3). We found a concomitant increase in fibroblast content in those same samples with decreased tumour purity at biopsy B (Additional file 2: Fig S4). No other cell types showed significant changes in abundance with chemotherapy treatment (Additional file 2: Fig S4).

To meet our primary aim of identifying associations between DNA methylation at diagnosis and NAC response, we focused our analysis on patient samples from the extreme end of response, namely pCR and nR, testing for global, probe-specific, and regional methylation differences. First, we used principal components analysis (PCA) to gain an overview of the association between DNA methylation measurements and known technical and clinical factors (Additional file 1). Each principal component (PC) is linearly independent and explains a particular proportion of the variance within the methylation data. PCs 1 and 2 captured 20.2% and 13.8% of the variance respectively and were significantly associated with patient age, EPIC chip, and cellular composition (Pearson correlation, *P* = 0.1 × 10^−5^–9.0 × 10^−5^). PC3, capturing 9.6% of the variation in the data, was significantly and singularly associated with NAC response (Pearson correlation, *P* = 0.03, Additional file 2: Fig S5a, b). Global methylation was interrogated through the proxy measure of methylation at targeted repeat regions of the genome including *LINE1, Alu* and *LTR*. Using a linear model with PC1 and 2 included as covariates, we observed no significant global methylation differences between responder groups (Additional file 2: Fig S6). Next, we sought to identify probe-specific methylation differences between pCR and nR using *limma* (adjusted for PC1 and 2 above, Additional file 2: Fig S5c, d). We identified 92 significantly differentially methylated probes (DMPs, *FDR* <  = 0.1, Additional file 3: Table S3), predominantly hypermethylated in non-responders. When hierarchically clustered, these probes show a well-defined discrimination between the two responder groups (Additional file 2: Fig S7a). Inclusion of the partial responders with the same DMPs clusters most partial responders with the non-responder patients (Additional file 2: Fig S7b).

Next, we sought to identify differentially methylated regions (DMRs, co-localised differentially methylated probes) between pCR and nR using *DMRcate*, with DMRs defined as having an absolute methylation difference (Δβ) greater than 10% and significance of *FDR* <  = 0.1 (adjusted for PC1 and 2). We identified 9 significant ‘*response-DMRs*’, overlapping RefSeq promoters (*TMEM176A/B*, *UNC5D, STAC2, SDR42E1, NELL1, GRP, FOXG1, CDH8, GRIA4)* and covering 7–14 CpG sites in each DMR. All 9 DMRs were hypermethylated in nR, with a Δβ value (nR-pCR) of 14–28% (Table 1, Fig. 1D). We correlated DNA methylation differences in the 9 response-DMRs with tumour purity at diagnosis and found no correlation (*r* ≤  ± 0.28, *P* = ns, Additional file 2: Fig S8), confirming that the methylation differences between responder and non-responder samples are independent of tumour purity. We further found no evidence of methylation change at these 9 DMRs before and after NAC (biopsies A vs B), suggesting that their methylation levels are unaffected by treatment (paired t-test, *P* > 0.05, Additional file 2: Fig S9). Finally, to ascertain whether the response-DMRs were capable of informing the degree of response to NAC we used receiver operating characteristic (ROC) analysis utilising only the pCR (*n* = 12) and partial responder samples (*n* = 13). Combining all 9 response-DMRs in the ROC analysis we observed an area under the curve of 0.891, showing that we can also distinguish complete from partial response to NAC with high sensitivity (Fig. 1E).

**Table 1:** Details of response-differentially methylated regions (response-DMRs)

Gene ID	Chr	Range (hg19)	Average Δβ (%)^α^	No. CpGs	*P*^β^	CpG Island % (Island/Shore)	RefSeq % (Promoter/Exon)	MCF7 ChromHMM % (Active/Bivalent/Polycomb/Quiescent)^γ^
*TMEM176A/B*	Chr7	150,497,065–150,498,205	27.2	14	2.04 × 10^−9^	100/0	90/10	0/0/0/100
*UNC5D*	Chr8	3,509,283–35,093,411	28.2	10	2.35 × 10^−4^	100/0	93/7	0/0/100/0
*STAC2*	Chr17	37,381,830–37,382,301	14.9	7	3.73 × 10^−4^	100/0	72/28	0/0/0/100
*SDR42E1*	Chr16	82,044,738–82,045,297	23.8	10	1.13 × 10^−3^	80/20	90/10	100/0/0/0
*NELL1*	Chr11	20,690,682–20,691,429	22.8	12	1.49 × 10^−3^	100/0	83/17	0/0/100/0
*GRP*	Chr18	56,887,002–56,887,785	20.3	11	2.97 × 10^−3^	73/27	73/27	0/0/100/0
*FOXG1*	Chr14	29,235,928–29,236,535	17.5	11	2.00 × 10^−2^	0/100	73/27	0/0/100/0
*CDH8*	Chr16	62,069,806–62,070,365	24.2	10	2.00 × 10^−2^	100/0	100/0	0/10/90/0
*GRIA4*	Chr11	105,480,979–105,481,322	15.4	7	2.26 × 10^−1^	86/14	86/14	0/0/100/0

^α^Calculated as Non-responders minus Complete responders

^β^Fisher’s multiple comparison statistic

^γ^Data from ENCODE MCF7 Segmentation (Additional file 1)

To determine whether the 9 hypermethylated response-DMRs overlapping gene promoters are associated with gene silencing of these genes in the nR group, we examined RNA-Seq expression data previously performed on the same SETUP diagnostic biopsy samples [7] (Additional file 1). We observed that non-responders with a hypermethylated *SDR42E1* promoter DMR (Fig. 1F) showed a significant decrease in expression of *SDR42E1* (Welch t-test, *P* = 0.025) compared to pCRs (Fig. 1G), with *TMEM176A and TMEM176B* showing a trend towards significance (Welch t-test, *P* = 0.054 and 0.073 respectively). We did not find any significant differences in expression in the other response-DMR genes, indeed the majority showed low levels of expression across all samples (Additional file 3: Table S5), consistent with their respective MCF7 bivalent/repressive ChromHMM states (Table 1) and concordant with TNBC TCGA expression (Additional file 2: Fig S10). We evaluated breast cancer single-cell RNA-seq data (*n* = 6 TNBC) [8] and found that the *SDR42E1* expression in our diagnostic biopsy samples is likely of epithelial cell origin while the *TMEM176A/B* expression is likely of stromal origin (Additional file 2: Fig S11).

We next used Cox proportional hazards models to assess the prognostic utility of known clinical variables and methylation in our cohort (partial responders included; *n* = 32). We show that patient response to NAC is associated with overall survival (Log-rank test, *P* = 0.024, Fig. 1H) [4], while other standard clinical details such as age and breast cancer stage did not yield any prognostic utility (Additional file 3: Table S6). We show that average methylation in 4/9 response-DMRs (*NELL1, GRP, CDH8, SDR42E1*) is significantly associated with overall survival (Cox proportional hazards model, HR = 1.56–1.70, *P* < 0.05, Fig. 1I and J, Additional file 3: Table S7).

## Discussion

The response of a tumour to NAC treatment in TNBC is highly variable and poorly understood. DNA methylation offers great potential as a biomarker of treatment response. Thus, we performed whole-genome methylation profiling in a TNBC NAC cohort to identify novel predictive DNA methylation biomarkers of NAC response.

We identified nine DMRs, significantly hypermethylated in non-responder patient samples, with the ability to distinguish both non-responders and partial responders from complete responders, although this is pending larger cohort validation, which is a limitation of this study. Notably, all nine response-DMR genes have been previously associated with cancer and cancer-related pathways (Additional file 3: Table S4 for details and references). Briefly, hypermethylation of *TMEM176A* is associated with metastasis and reduced overall survival in colorectal cancer while *UNC5D* is a novel putative metastatic suppressor gene shown to be commonly hypermethylated in prostate cancer. Both *STAC2* and *NELL1* promoter CpG island hypermethylation has been reported in metastatic breast cancer and primary colon cancer, respectively and *GRP* is implicated in the development of tumorigenicity and drug resistance. *FOXG1* is an evolutionarily conserved forkhead-box transcriptional co-repressor with low levels shown to be associated with poor prognosis in breast cancer. *CDH8* codes for an integral membrane protein from the cadherin family where loss of *CDH8* has been reported in breast carcinoma. *GRIA4* hypermethylation has previously been reported to have prognostic value in breast cancer. Interestingly, *NELL1* overexpression is associated with chemotherapeutic sensitivity to cis/carboplatin and reduced colony formation in lung cancer, suggesting that epigenetic regulation of this gene may be important in chemotherapy response. Finally, *SDR42E1* involved in oxidoreductase activity, is the only DMR that shows both altered methylation and expression in this study. Liu et al*.* has previously shown that *SDR42E1* methylation is associated with differential gene expression in *TET* mutated diffuse large B-cell lymphoma [9], highlighting a potentially important role in cancer and making it a key gene for future functional studies.

Typically, pathological complete response to NAC correlates with better clinical outcomes, while residual disease after NAC is associated with higher risk of relapse and poorer survival among TNBC patients. Indeed, we show that the 9 DMRs associated with TNBC SETUP biopsy response to NAC, also have significant prognostic value in the SETUP patients. Pineda et al. [10] also reported a two gene epigenetic signature (*FERD3L* and *TRIP10*) for prediction of response to NAC in TNBC. There is no overlap in the response-DMR biomarkers identified in our two studies, likely due to the discordant neoadjuvant chemotherapy regimens used in each of the discovery cohorts. Nonetheless these studies each demonstrate the value of epigenetic signatures for predicting response to NAC and further exemplify the clinical interest and need for an all-encompassing NAC predictive methylation panel.

Overall, we have shown the potential of DNA methylation to be used as a predictive biomarker of response to NAC in TNBC. The utility of these biomarkers for predicting treatment response and long-term prognostic outcome in TNBC requires validation on larger cohorts and importantly, developing tailor-made biomarkers to specific NAC regimes may be warranted.

## Supplementary Information

**Additional file 1.** Extended Methods

**Additional file 2. Figure S1** Samples cluster by patient according to SNP probes as expected. Non-hierarchical clustering of beta values of 59 SNP quality control probes. Dendrogram coloured by the top 8 clusters found within the data. **Figure S2** Tumour purity is not different between responder groups at diagnosis. **a)** *InfiniumPurify* and *EpiDISH* purity estimations are highly correlated (Pearson’s correlation) **b)** Estimated epithelial content for patients grouped by responder status at each timepoint. Dashed line represents median for all samples at each timepoint. **Figure S3** All samples exhibit heterogeneous cellular composition. *EpiDISH* estimated cellular composition of each TNBC sample (*n* = 59, normal samples excluded). Cell types in order of inclusion: Epithelial, natural killer, neutrophils, monocytes, fibroblasts, eosinophils, CD8 + T-cells, CD4 + T-cells, and B-cells. **Figure S4** Reduction in tumour purity after 12 weeks of treatment in complete and partial responders but not in non-responders. *EpiDISH* estimated cellular composition in matched patient biopsy samples at diagnosis (A) and mid-way through NAC (B) (paired t-test). **Figure S5** Variance in DNA methylation at diagnosis associated with response to NAC. Principal components analysis (PCA) shows a) Pearson’s correlation (top = r, bottom = *P*) between PC eigenvector and known clinical and technical variables, and b) the percentage of variance in the methylation data explained by the top 5 PCs. After identification of PC1 and PC2 as being associated with technical and biological variables other than the variable of interest (response), we demonstrate that after removal of PC1 and PC2 using the removeBatcheffects function, a new PC analysis showed that variation in methylation associated with these variables is removed (c, d). This supports our inclusion of PC1 and PC2 in our linear model to identify response-DMRs. **Figure S6** No difference in global methylation between complete responders and non-responders at diagnosis. Global methylation estimated from EPIC array probes overlapping repetitive elements (RE) *LINE1, Alu and LTR*. **Figure S7** Methylation at 92 significant DMPs clusters patients by response to NAC. Dendrogram and heatmap of methylation of the 92 significant DMPs in samples from a) responders (*n* = 12) and non-responders (*n* = 7), and b) with partial responders (*n* = 13) included. **Figure S8** Correlation of DNA methylation at response-DMRs with InfiniumPurify derived tumour purity measures. Methylation levels of 9 significant response-DMRs correlated with the InfiniumPurify derived tumour purity shows no correlation between these measures (*r* ≤  ± 0.28, *P* = ns). Points are coloured by response. **Figure S9** DNA methylation at response-DMRs is stable between diagnosis and mid-NAC. Methylation levels at the 9 DMRs between biopsies A and B, separated into complete responders and non-responders (paired t-test). **Figure S10** Low levels of gene expression at genes with promoter overlapping response-DMRs in the TCGA TNBC dataset. RNA-seq expression levels Log2(TPM + 1). **Figure S11** In breast tumour tissue *SDR42E1* is localised to the epithelial cells, whilst *TMEM176A/B* is localised to stromal cells. Single-cell breast cancer data from 6 patients showing expression of the 10 genes proximal to the 9 response-DMRs. Size of dot is proportional to the percentage of cells in that cell type expressing that gene and the colour represents the scaled mean expression of that gene within the cell type

**Additional file 3.** Supplementary Tables

## Data Availability

The datasets generated and analysed during the current study are publicly available at NCBI GEO (www.ncbi.nlm.nih.gov/geo) under accession numbers GSE184159.
