# Supplementary material for: Identification of DNA methylation biomarkers with potential to predict response to neoadjuvant chemotherapy in triple-negative breast cancer
Source: Clin Epigenetics. 2021 Dec 18;13:226. doi: 10.1186/s13148-021-01210-6 (PMC8684655; doi:10.1186/s13148-021-01210-6)
Supplement: Supplementary file 1 — Additional file 1. Extended Methods [file 13148_2021_1210_MOESM1_ESM.docx]

**Supplemental Information**

**Additional Methods**

**Clinical samples**

Sequential Evaluation of Tumours Undergoing Preoperative chemotherapy (SETUP) study triple-negative breast cancer samples were obtained from Monash and Peninsula Health (Melbourne, Australia). All patients enrolled in the trial received 12 weeks of fluorouracil, epirubicin, and cyclophosphamide, and three months of docetaxel. Tumour size was monitored via PET imaging and CT scanning. The primary endpoint of this study was pathological response at time of surgical resection. Patients were considered complete responders where there was no evidence of tumour remaining post chemotherapy (confirmed by PET, CT, and histology), partial responders where there was a reduction in tumour size from baseline, and non-responders where tumour size was unchanged or progressed through chemotherapy (Table S1).

**Quality control and pre-processing of Illumina EPIC array data**

DNA (500 ng) was treated with sodium bisulphite using the EZ-96 DNA methylation kit (Zymo Research, CA, USA).

Raw intensity data (IDAT) files were imported into the R environment (version 3.1.1) using the *minfi* package (version 1.32.0) (1). Data quality and grouping of patient-matched samples was checked with plots derived from control probes on the array. Data was then normalised with *preprocessFunnorm*. Each sample passed all quality control steps; at least 99% of probes had detection *P* values >0.01. Poor quality probes with a detection *P* value >0.01 in at least 10% samples were removed. Probes mapping to multiple locations or overlapping SNPs were discarded to reduce the risk of false discoveries, as previously described (2)

β values were calculated from unmethylated (U) and methylated (M) signal [M/(U + M + 100)] and ranged from 0 to 1 (0 to 100% methylation). The co-ordinates of all CpG sites were defined using the hg19 human genome assembly.

**Cellular deconvolution**

The ‘*InfiniumPurify’* (3) R package was used to estimate tumour purity from the processed DNA methylation data. The ‘*EPIDish’* (4) package was used to validate this prediction and estimate the proportion of eight further cell types in each sample – Natural killer cells, neutrophils, monocytes, fibroblasts, eosinophils, CD8+ T-cells, CD4+ T-cells, and B-cells. Statistical analyses (Welch’s t-test and paired t-test) were undertaken to determine if tumour purity or proportion of other cell types differed between responder groups.

**Microarray genome-wide DNA methylation analysis**

For initial mining of the EPIC data, principal components analysis was undertaken using the ‘*prcomp’* (‘stats’ base R package) (1). PC1 and PC2 were identified as being associated with technical and biological variables other than the variable of interest in this study (response). Using the ‘*removeBatchEffect*’ function from the *limma* package, we are able to demonstrate (through a new principal components analysis) how this removes the unwanted variation associated with PC1 and PC2, leaving the remaining major source of variation associated with patient NAC response. For all further analyses we used PC1 and PC2 as covariates.

We used the *limma* package to identify differentially methylated probes (DMPs) between these response groups with adjusted p-value cut-off of FDR<0.1. DMPs were visualised as heatmaps using the ‘*ComplexHeatmap’* (5) R package (version 2.2.0). The R package *DMRcate* was used to identify differentially methylated regions (DMRs) i.e., response-DMRs, with a DMR p-value cut-off of FDR<0.1 and a ∆β of >=10%. DMRs were defined as regions with a maximum of 1000 nucleotides between consecutive probes, a minimum of 2 CpG sites. Fisher’s multiple comparison statistic was used to rank significant DMRs. BedGraph files of the methylation data were generated for visualisation in the IGV genome browser. Correlation of methylation findings with tumour purity was undertaken to ensure that tumour purity was not a confounding factor.

We downloaded ChIP-seq data from the ENCODE portal (ENCSR761DLU, ENCSR999WHE, ENCSR985MIB, ENCSR610IYQ, ENCSR493NBY) and examined the overlap of our DMRs with the respective chromatin states in MCF7 cells (6, 7).

**Profiling expression using RNA-Seq**

RNA-Seq data for all samples (n=32) was obtained upon request from Brockwell *et al* (8). 125bp paired-end reads were checked for quality and processed using FASTQC version 0.11.5 (github.com/s-andrews/FastQC), and TrimGalore version 0.5 (github.com/FelixKrueger/TrimGalore). High-quality reads were then mapped to the human reference genome GRCh38.p13 (ftp://ftp.ebi.ac.uk/pub/databases/gencode/Gencode_human/release_19/GRCh38.p13.genome.fa.gz) and Gencode gene annotation release 32 (ftp://ftp.ebi.ac.uk/pub/databases/gencode/Gencode_human/release_32/gencode.v32.annotation.gtf.gz) using STAR version 2.7.0e (9). Mapped reads on the transcriptomes were sorted by novosort version 1.03.08 (10). Gene expression levels were quantified in transcripts per million (TPM) and raw count by RSEM version 1.3.1 (11).

Single cell expression profiling was obtained from Wu *et al* (12), where we imported the list of 10 genes overlapping the 9 response-DMRs and exported cell-type specific expression across 6 patient tumours as a dot-plot (https://singlecell.broadinstitute.org/single_cell/study/SCP1106/stromal-cell-diversity-associated-with-immune-evasion-in-human-triple-negative-breast-cancer).

**Survival analysis**

Survival analysis was undertaken using functions in the *survival* package version 2.39 (13). A log-rank test was used to examine the association between patient response to chemotherapy and overall survival and visualised using a Kaplan-Meier plot. Cox proportional hazard models were used to assess the association between DNA methylation at each response-DMR and overall survival, with principal components 1 and 2 used as covariates. Results were visualised using Kaplan-Meier plots and log-rank p-value with patients stratified by methylation of samples in the top 25% or bottom 75%.

**Packages used**

| **Package** | **Reference** | **Package** | **Reference** |
| --- | --- | --- | --- |
| *R Environment* | (14) | *Conumee* | (15) |
| *Minfi* | (1) | *ComplexHeatmap* | (5) |
| *Limma* | (16) | *DMRcate* | (17) |
| *InfiniumPurify* | (3) | *REMP* | (18) |
| *EPIdish* | (4) | *survival* | (13) |

**References**

1. Aryee MJ, Jaffe AE, Corrada-Bravo H, Ladd-Acosta C, Feinberg AP, Hansen KD, et al. Minfi: a flexible and comprehensive Bioconductor package for the analysis of Infinium DNA methylation microarrays. Bioinformatics. 2014;30(10):1363-9.

2. Pidsley R, Zotenko E, Peters TJ, Lawrence MG, Risbridger GP, Molloy P, et al. Critical evaluation of the Illumina MethylationEPIC BeadChip microarray for whole-genome DNA methylation profiling. Genome Biology. 2016;17(1):208.

3. Qin Y, Feng H, Chen M, Wu H, Zheng X. InfiniumPurify: An R package for estimating and accounting for tumor purity in cancer methylation research. Genes Dis. 2018;5(1):43-5.

4. Zheng SC, Breeze CE, Beck S, Dong D, Zhu T, Ma L, et al. EpiDISH web server: Epigenetic Dissection of Intra-Sample-Heterogeneity with online GUI. Bioinformatics. 2020;36(6):1950-1.

5. Gu Z, Eils R, Schlesner M. Complex heatmaps reveal patterns and correlations in multidimensional genomic data. Bioinformatics. 2016;32(18):2847-9.

6. An integrated encyclopedia of DNA elements in the human genome. Nature. 2012;489(7414):57-74.

7. Davis CA, Hitz BC, Sloan CA, Chan ET, Davidson JM, Gabdank I, et al. The Encyclopedia of DNA elements (ENCODE): data portal update. Nucleic Acids Res. 2018;46(D1):D794-d801.

8. Brockwell NK, Rautela J, Owen KL, Gearing LJ, Deb S, Harvey K, et al. Tumor inherent interferon regulators as biomarkers of long-term chemotherapeutic response in TNBC. NPJ Precis Oncol. 2019;3:21.

9. Dobin A, Davis CA, Schlesinger F, Drenkow J, Zaleski C, Jha S, et al. STAR: ultrafast universal RNA-seq aligner. Bioinformatics. 2013;29(1):15-21.

10. BHD NTS. Novosort 2014 [Available from: <http://www.novocraft.com/>.

11. Li B, Dewey CN. RSEM: accurate transcript quantification from RNA-Seq data with or without a reference genome. BMC Bioinformatics. 2011;12(1):323.

12. Wu SZ, Roden DL, Wang C, Holliday H, Harvey K, Cazet AS, et al. Stromal cell diversity associated with immune evasion in human triple-negative breast cancer. The EMBO Journal. 2020;39(19):e104063.

13. Grambsch TMTaPM. Modeling Survival Data: Extending the Cox Model: Springer; 2000.

14. Team RC. R: A language and environment for statistical computing. Vienna, Austria; 2013.

15. Volker Hovestadt MZ. conumee: Enhanced copy-number variation analysis using Illumina DNA methylation arrays. R package version 190 <URL: <http://bioconductororg/packages/conumee/>>. 2017.

16. Ritchie ME, Phipson B, Wu D, Hu Y, Law CW, Shi W, et al. limma powers differential expression analyses for RNA-sequencing and microarray studies. Nucleic Acids Res. 2015;43(7):e47-e.

17. Peters TJ, Buckley MJ, Statham AL, Pidsley R, Samaras K, R VL, et al. De novo identification of differentially methylated regions in the human genome. Epigenetics & chromatin. 2015;8:6.

18. Zheng Y, Joyce BT, Liu L, Zhang Z, Kibbe WA, Zhang W, et al. Prediction of genome-wide DNA methylation in repetitive elements. Nucleic Acids Res. 2017;45(15):8697-711.
