# Supplementary material for: Identification of DNA methylation biomarkers with potential to predict response to neoadjuvant chemotherapy in triple-negative breast cancer
Source: Clin Epigenetics. 2021 Dec 18;13:226. doi: 10.1186/s13148-021-01210-6 (PMC8684655; doi:10.1186/s13148-021-01210-6)

Supplementary Figure 1

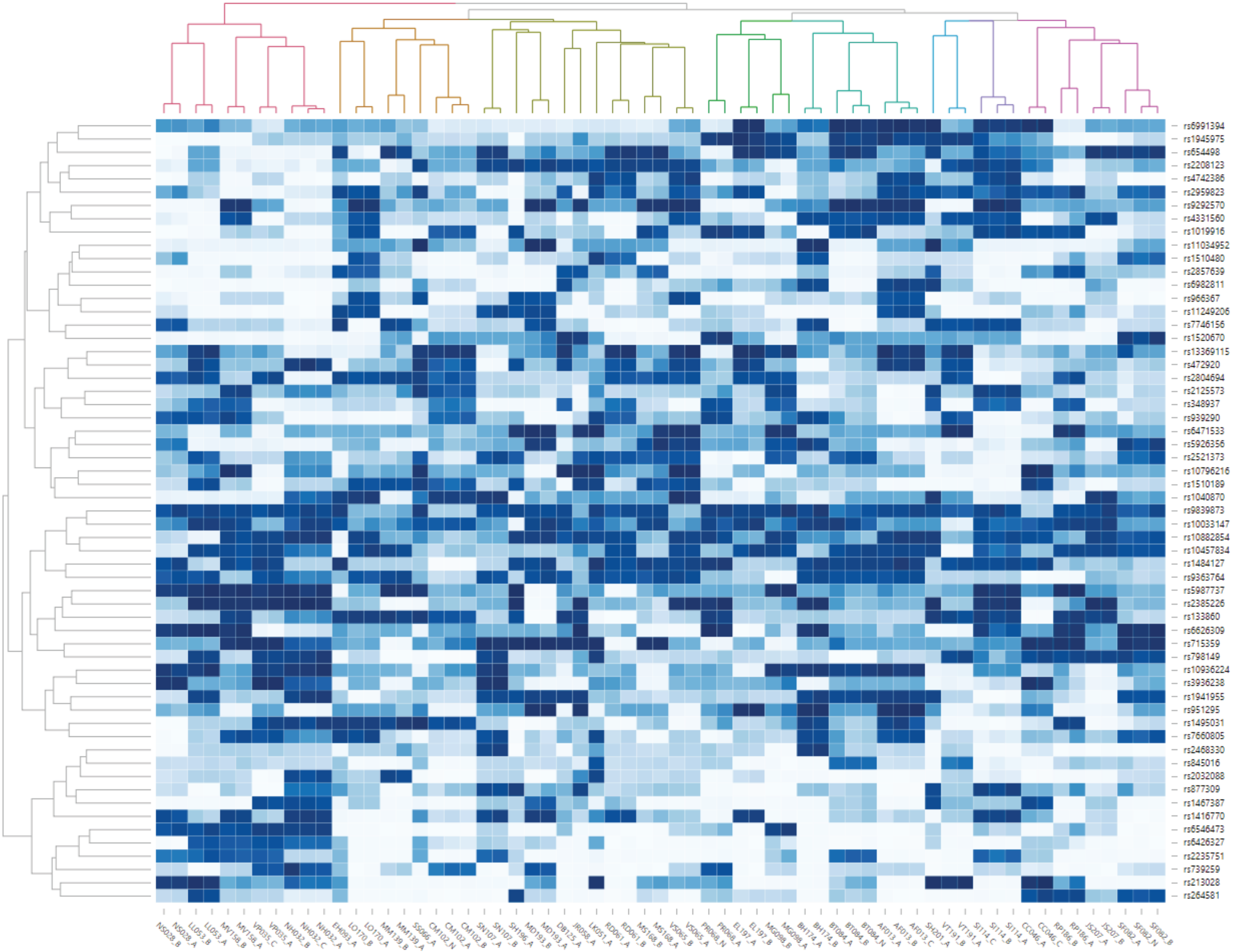

Supplementary Figure 2a

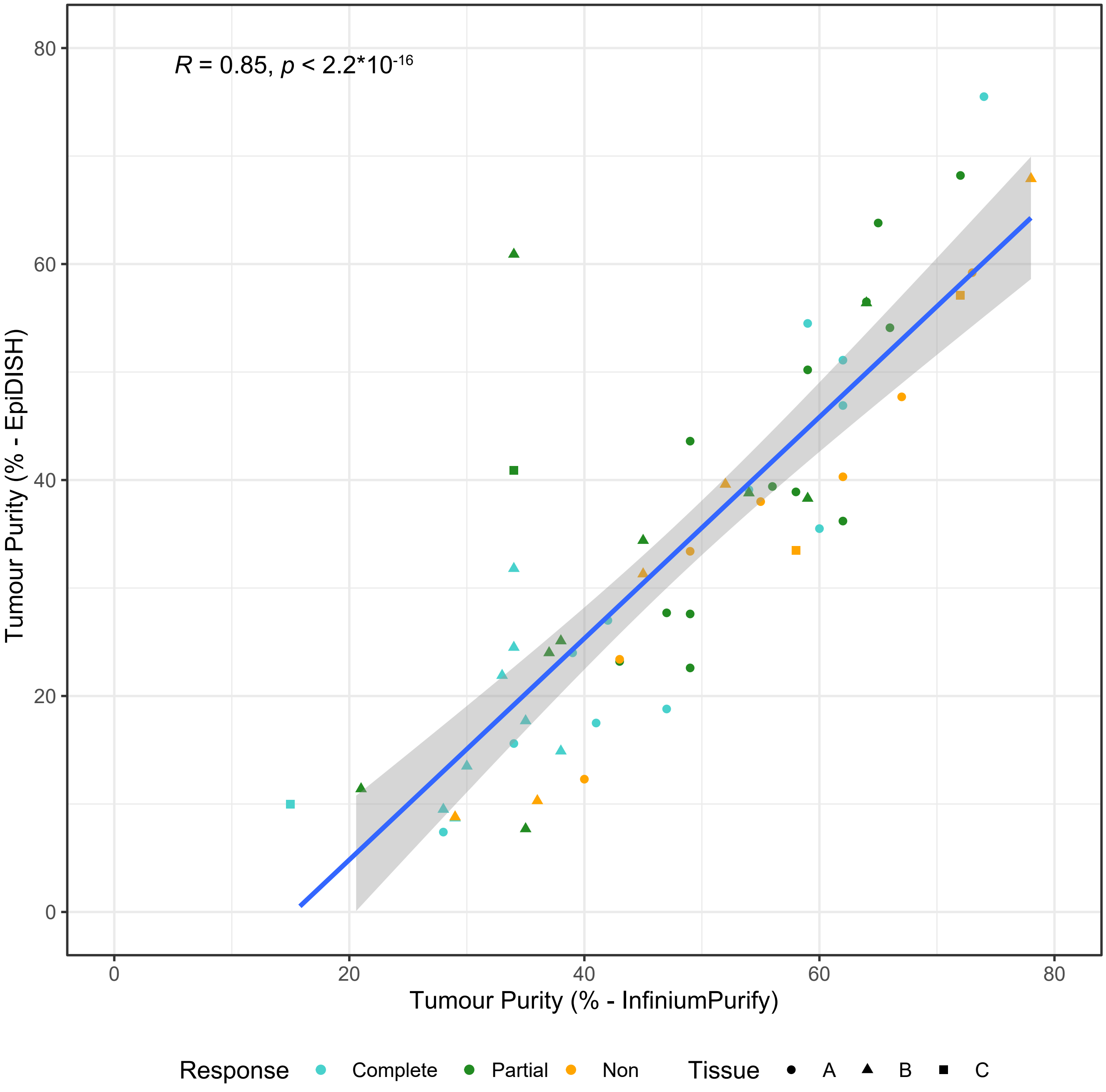

Supplementary Figure 2b

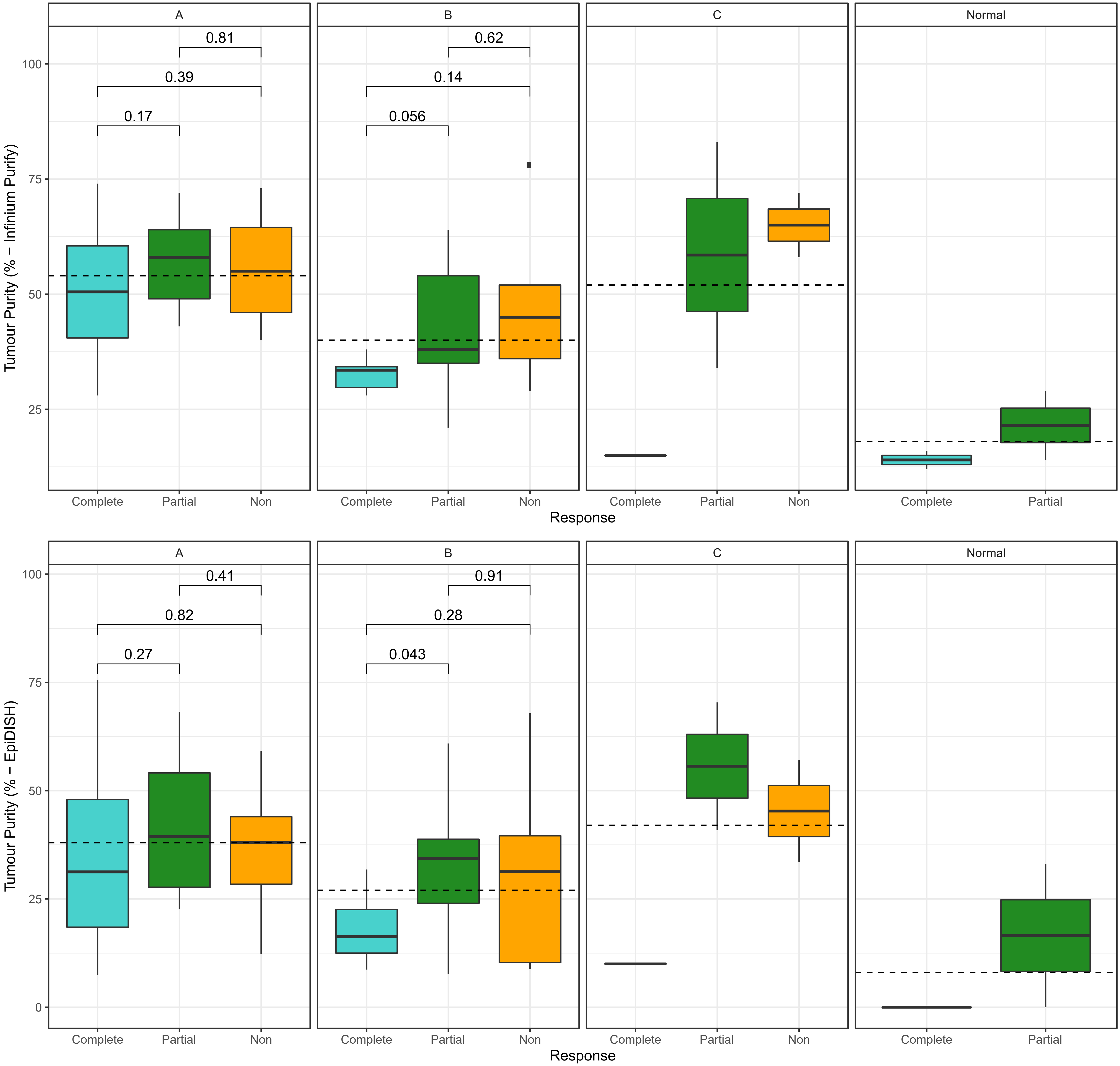

Supplementary Figure 3

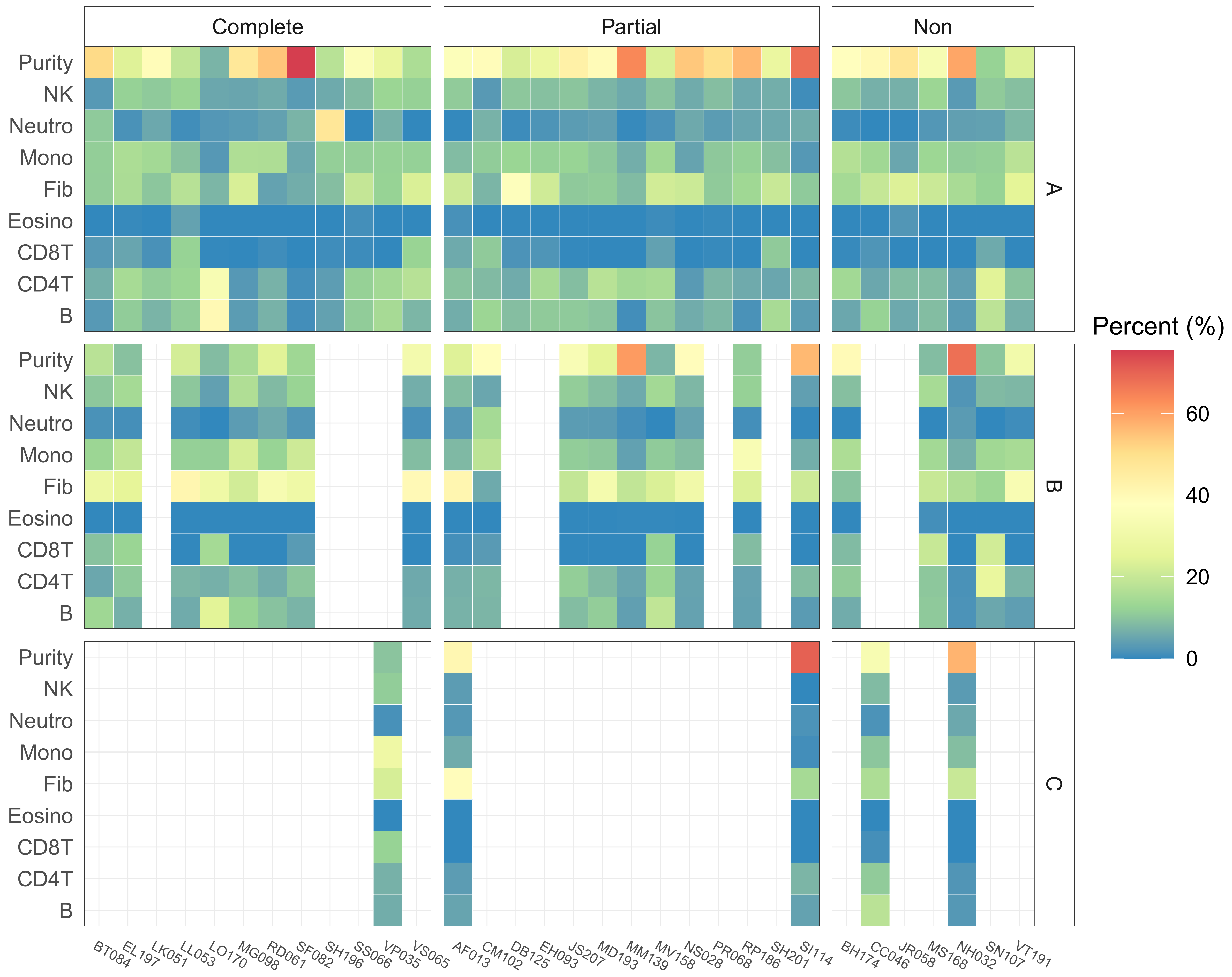

Supplementary Figure 4

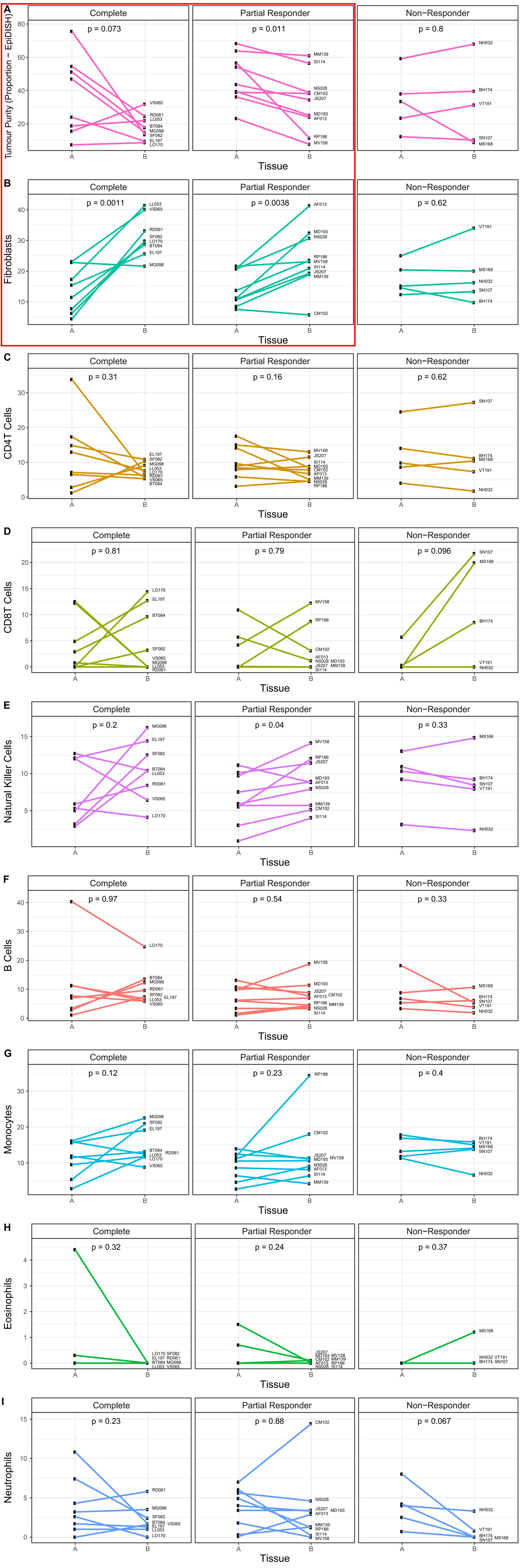

Supplementary Figure 5

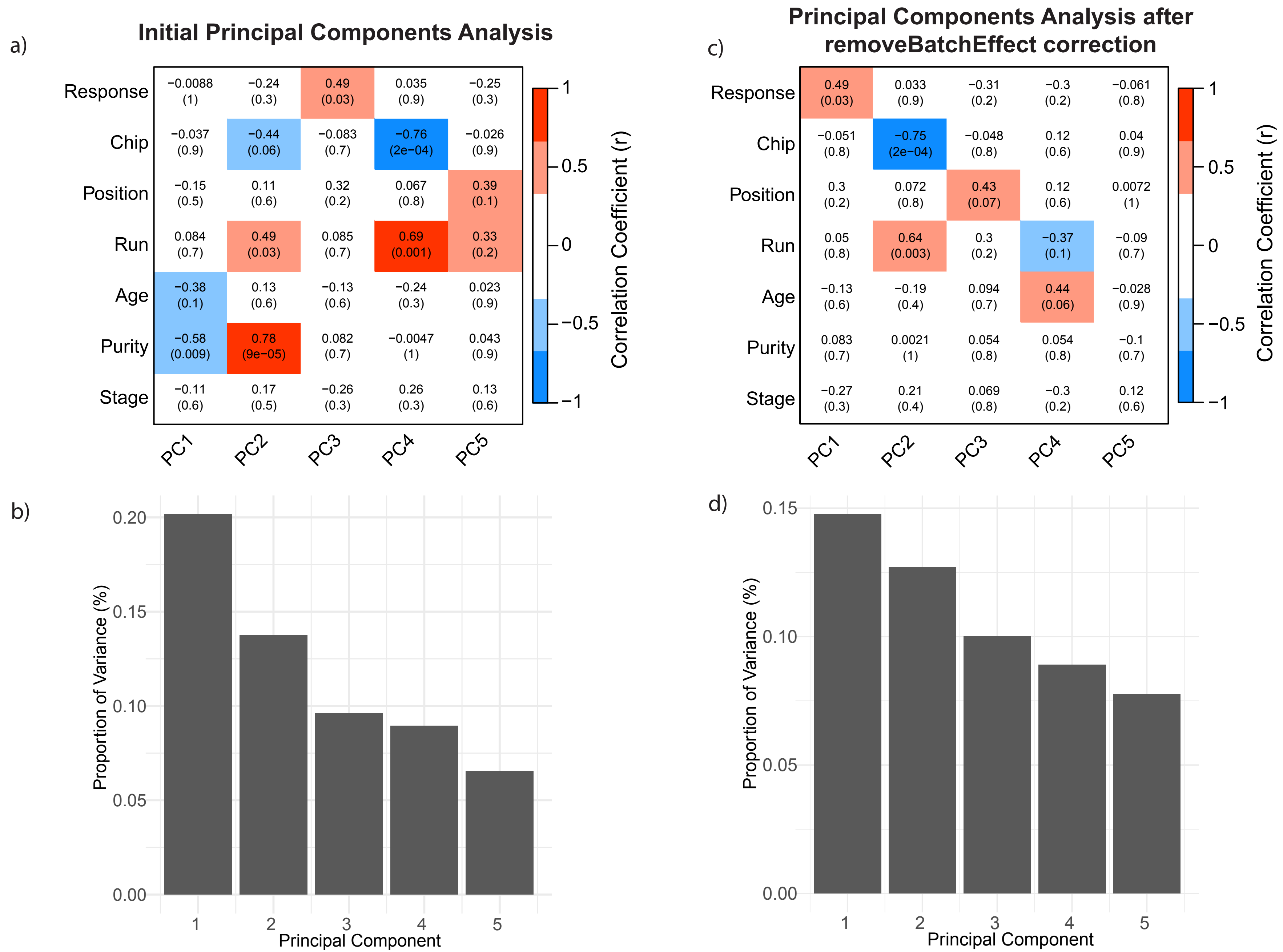

Supplementary Figure 6

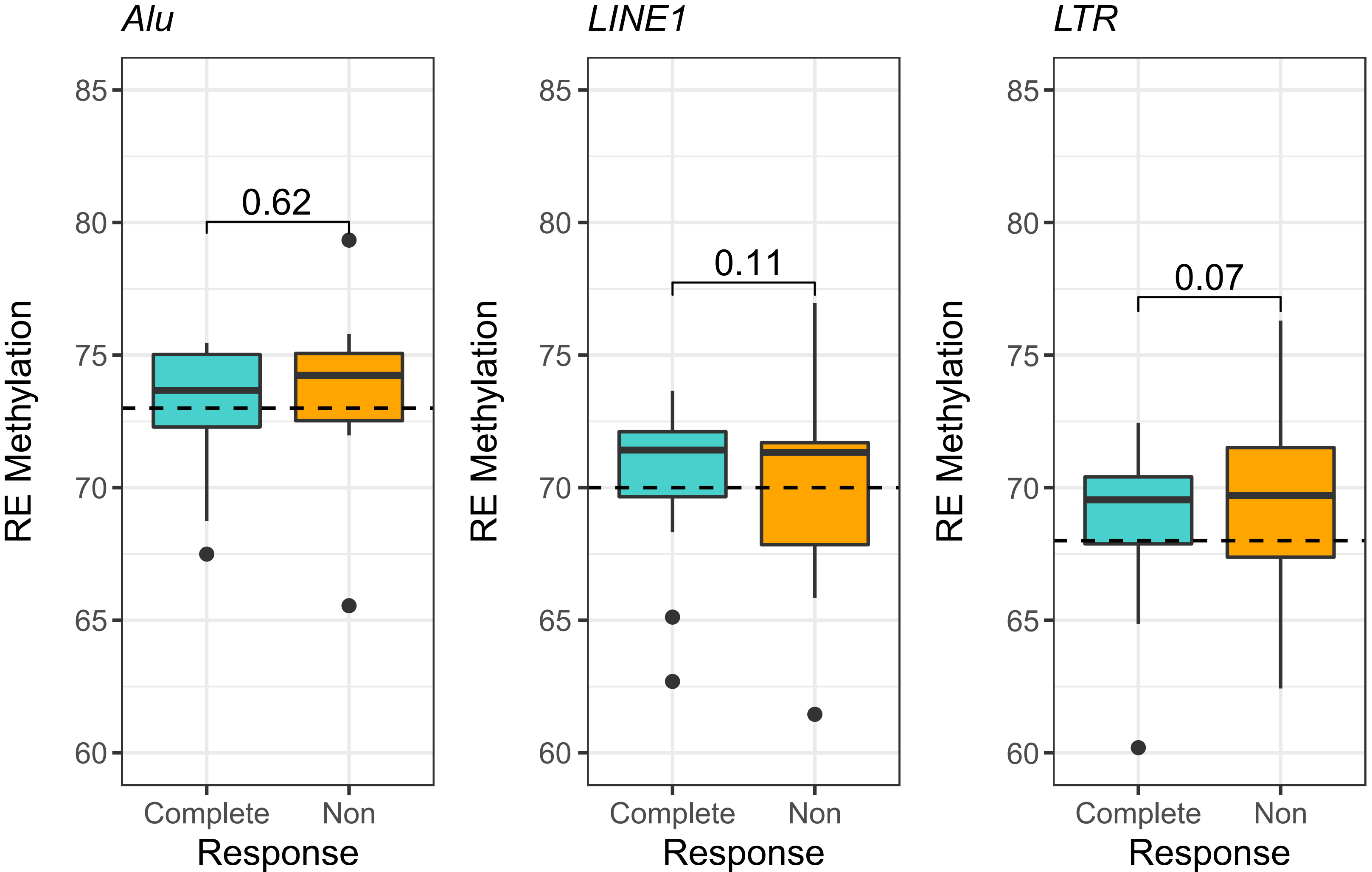

Supplementary Figure 7a

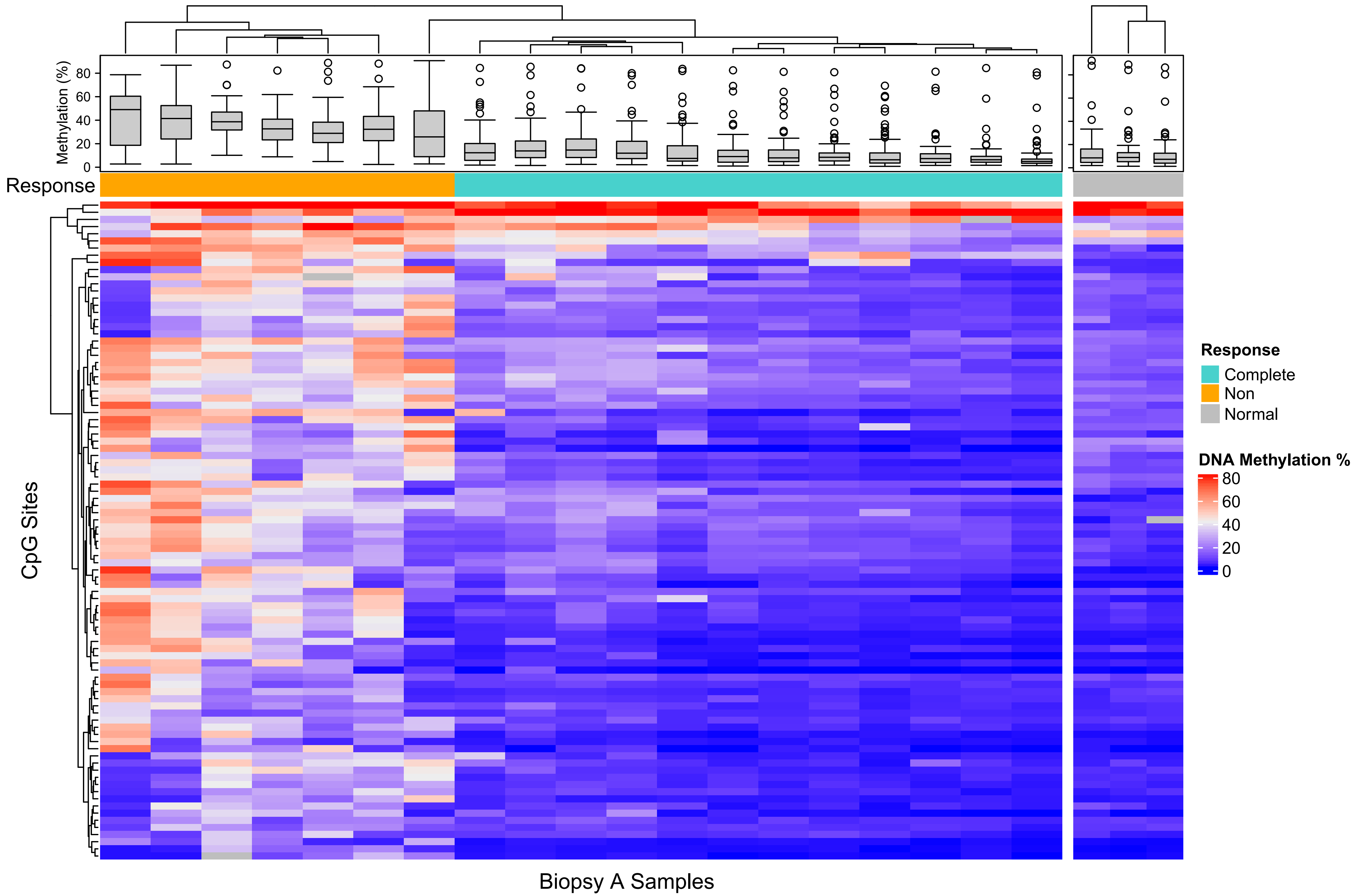

Supplementary Figure 7b

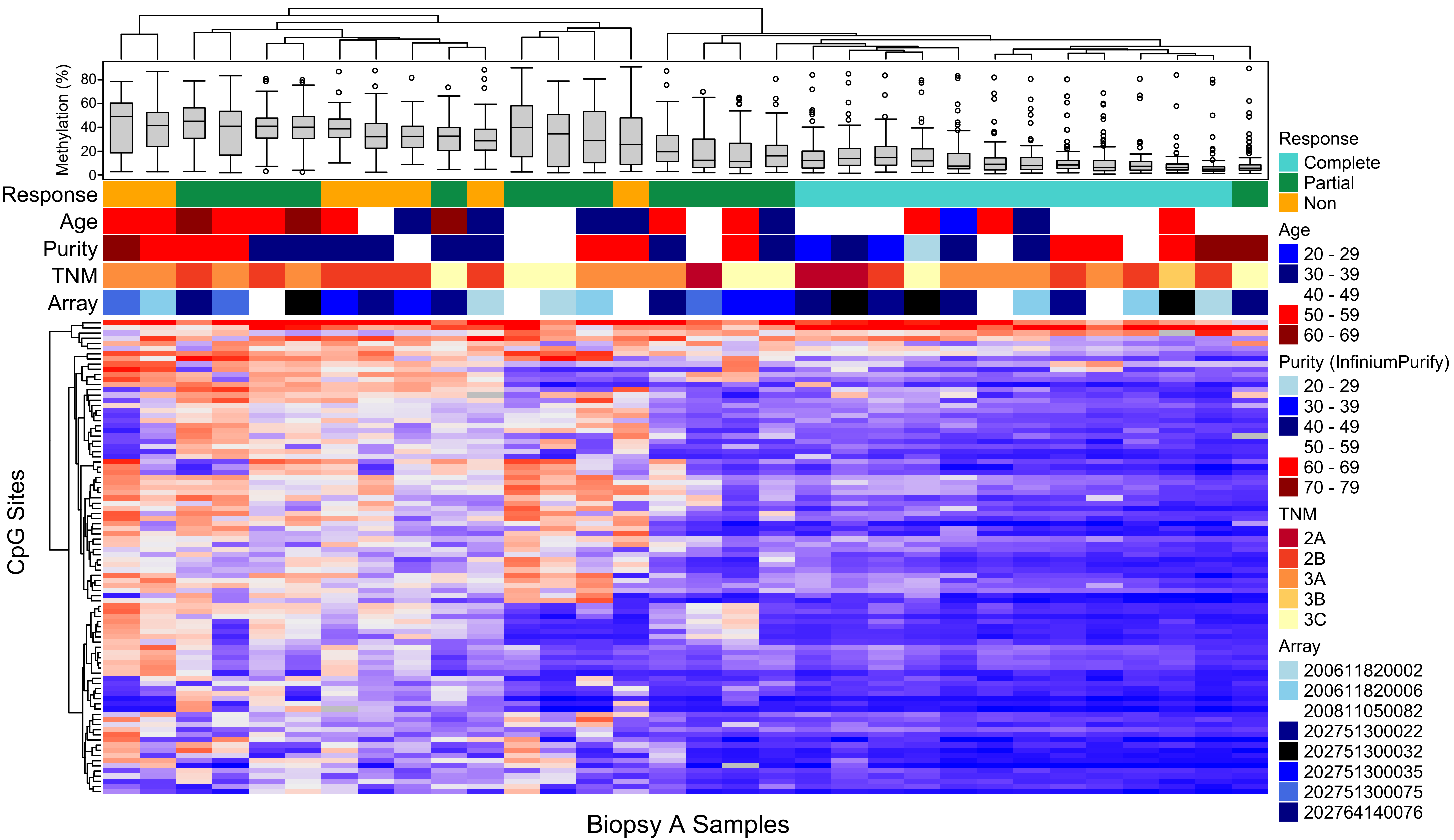

Supplementary Figure 8

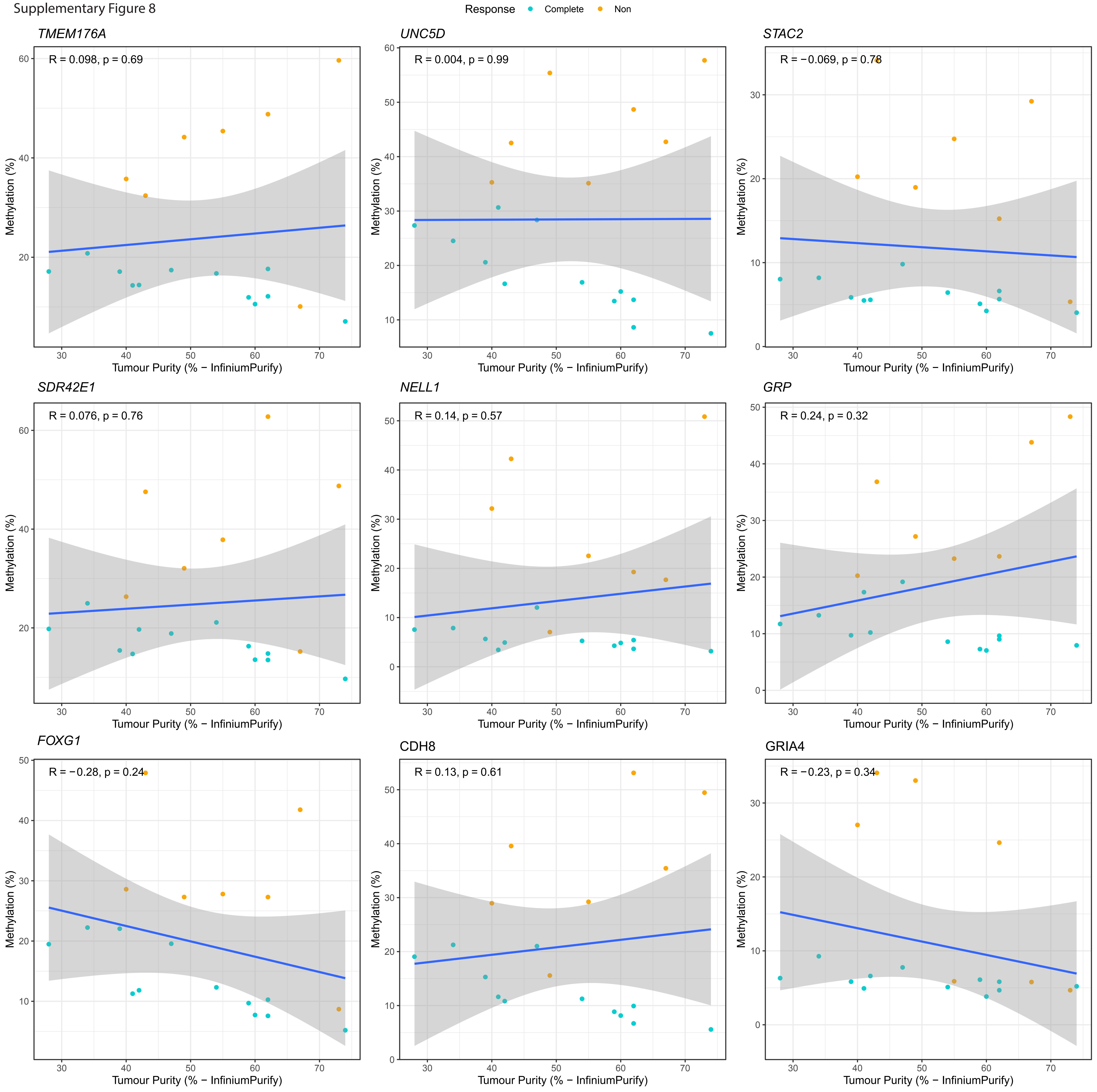

Supplementary Figure 9

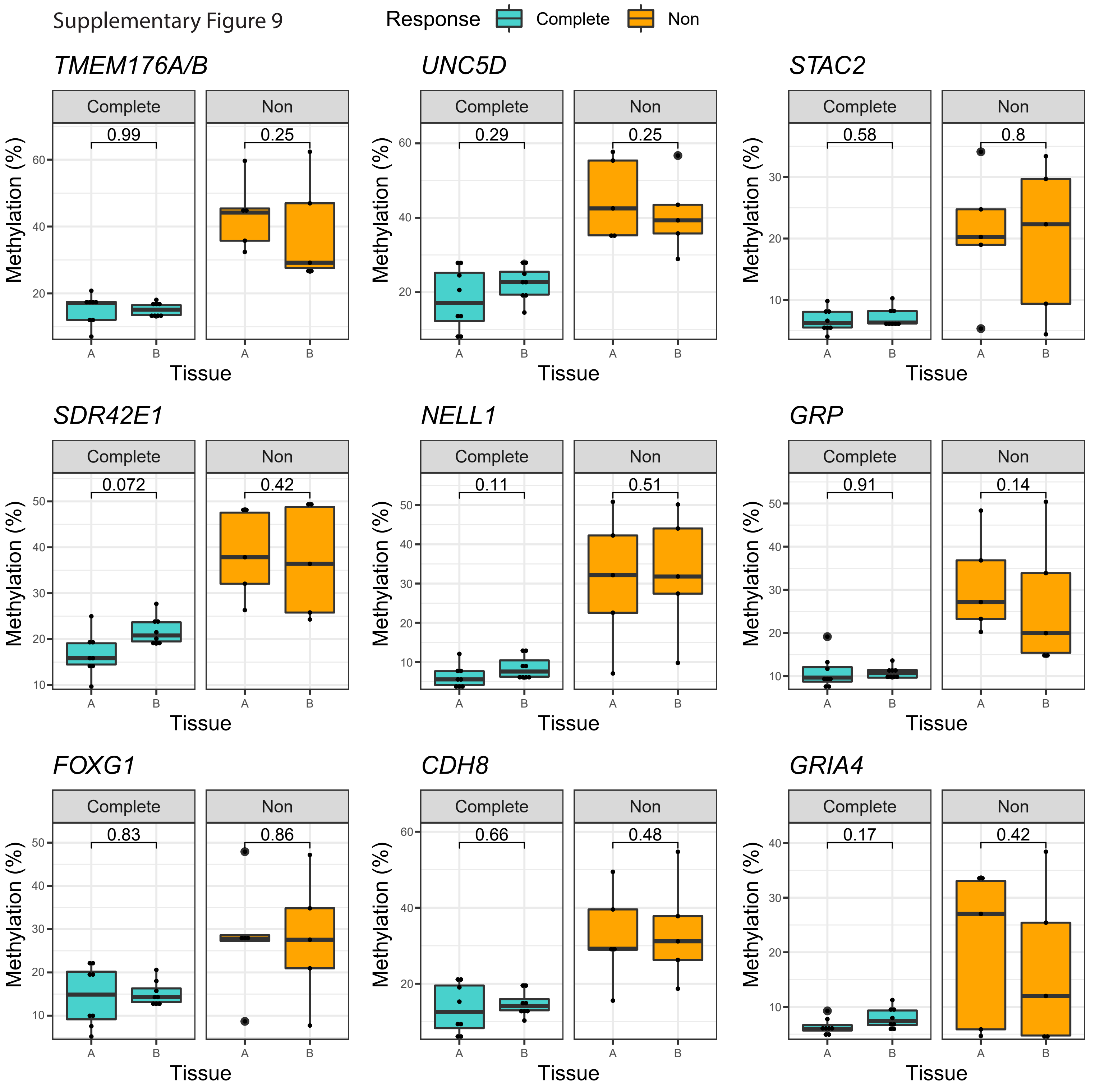

Supplementary Figure 10

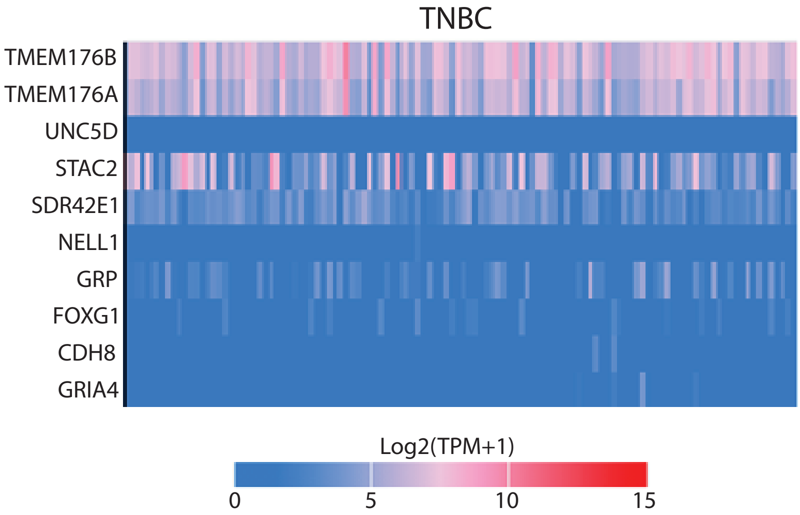

Supplementary Figure 11

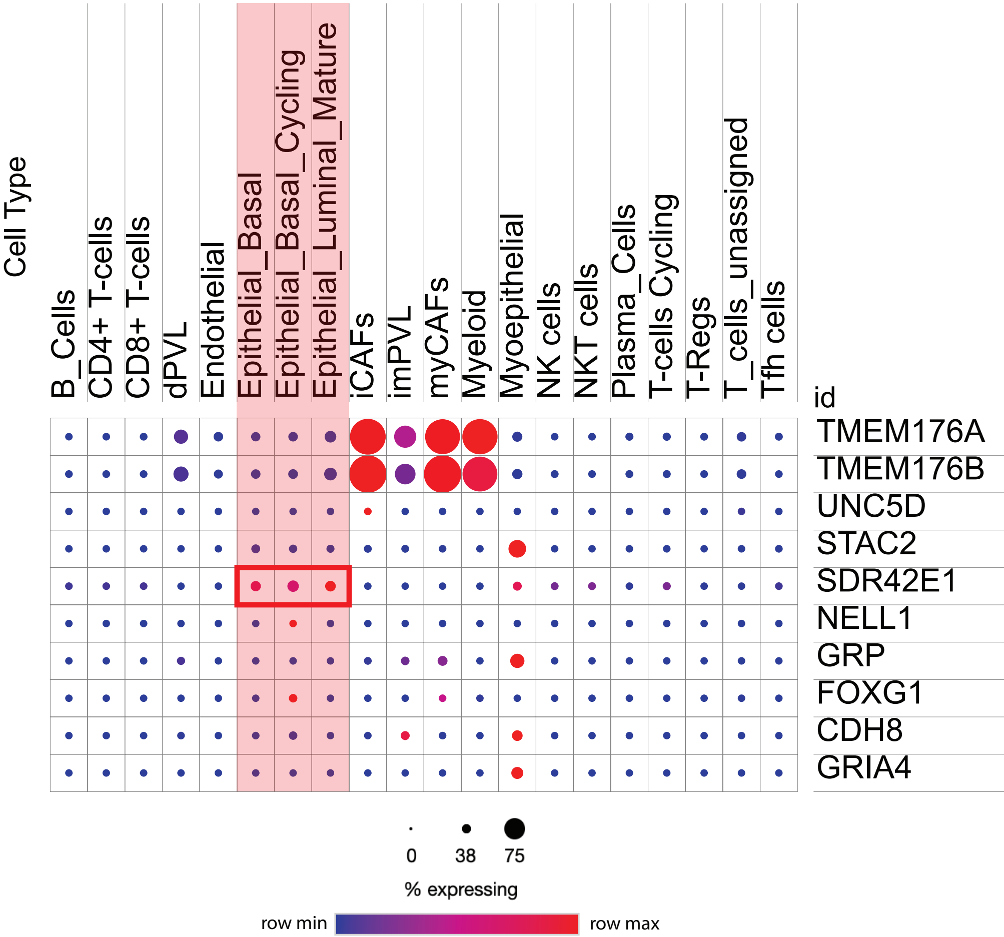

Supplement: Supplementary file 2 — Additional file 2. Figure S1 Samples cluster by patient according to SNP probes as expected. Non-hierarchical clustering of beta values of 59 SNP quality control probes. Dendrogram coloured by the top 8 clusters found within the data. Figure S2 Tumour purity is not different between responder groups at diagnosis. a) InfiniumPurify and EpiDISH purity estimations are highly correlated (Pearson’s correlation) b) Estimated epithelial content for patients grouped by responder status at each timepoint. Dashed line represents median for all samples at each timepoint. Figure S3 All samples exhibit heterogeneous cellular composition. EpiDISH estimated cellular composition of each TNBC sample (n = 59, normal samples excluded). Cell types in order of inclusion: Epithelial, natural killer, neutrophils, monocytes, fibroblasts, eosinophils, CD8 + T-cells, CD4 + T-cells, and B-cells. Figure S4 Reduction in tumour purity after 12 weeks of treatment in complete and partial responders but not in non-responders. EpiDISH estimated cellular composition in matched patient biopsy samples at diagnosis (A) and mid-way through NAC (B) (paired t-test). Figure S5 Variance in DNA methylation at diagnosis associated with response to NAC. Principal components analysis (PCA) shows a) Pearson’s correlation (top = r, bottom = P) between PC eigenvector and known clinical and technical variables, and b) the percentage of variance in the methylation data explained by the top 5 PCs. After identification of PC1 and PC2 as being associated with technical and biological variables other than the variable of interest (response), we demonstrate that after removal of PC1 and PC2 using the removeBatcheffects function, a new PC analysis showed that variation in methylation associated with these variables is removed (c, d). This supports our inclusion of PC1 and PC2 in our linear model to identify response-DMRs. Figure S6 No difference in global methylation between complete responders and non-responders at diagno [file 13148_2021_1210_MOESM2_ESM.pdf]
